# Synergy-COPD: a systems approach for understanding and managing chronic diseases

**DOI:** 10.1186/1479-5876-12-S2-S2

**Published:** 2014-11-28

**Authors:** David Gomez-Cabrero, Magi Lluch-Ariet, Jesper Tegnér, Marta Cascante, Felip Miralles, Josep Roca, the Synergy-COPD consortium

**Affiliations:** 1 Unit of Computational Medicine, Department of Medicine, Center for Molecular Medicine, Karolinska Institutet, Karolinska University Hospital, Stockholm, Sweden; 2 Department of eHealth, Barcelona Digital, 08017 Barcelona, Catalunya, Spain; 3 Hospital Clinic - Institut d'Investigacions Biomediques August Pi i Sunyer (IDIBAPS). Universitat de Barcelona, 08036 Barcelona, Spain; 4 Departament de Bioquimica i Biologia Molecular i IBUB, Facultat de Biologia, Universitat de Barcelona, 08028 Barcelona, Spain; 5 Centro de Investigación Biomédica en Red de Enfermedades Respiratorias (CIBERES), Bunyola, Balearic Islands

**Keywords:** Chronic Diseases, Chronic Obstructive Pulmonary Disease, COPD, Integrated Care, ICT, Systems Medicine, Predictive Medicine, Telemedicine

## Abstract

Chronic diseases (CD) are generating a dramatic societal burden worldwide that is expected to persist over the next decades. The challenges posed by the epidemics of CD have triggered a novel health paradigm with major consequences on the traditional concept of disease and with a profound impact on key aspects of healthcare systems.

We hypothesized that the development of a systems approach to understand CD together with the generation of an ecosystem to transfer the acquired knowledge into the novel healthcare scenario may contribute to a cost-effective enhancement of health outcomes. To this end, we designed the Synergy-COPD project wherein the heterogeneity of chronic obstructive pulmonary disease (COPD) was addressed as a use case representative of CD.

The current manuscript describes main features of the project design and the strategies put in place for its development, as well the expected outcomes during the project life-span. Moreover, the manuscript serves as introductory and unifying chapter of the different papers associated to the Supplement describing the characteristics, tools and the objectives of Synergy-COPD

## Introduction

### The challenge of chronic diseases

Population ageing and changes in life-style are the two major factors explaining the dramatic increase in the prevalence of chronic diseases (CD) over the second half of the 20^th ^Century [1,2], as well as the prospects for the next decades [3]. It is widely accepted that CD represent 77% of the total disease burden on healthcare with relevant deleterious consequences on both disability and mortality [1-3]. The high societal impact of the epidemics of CD triggered the 2011 General Assembly of the United Nations convened to generate policies aiming at facing the challenges imposed by the phenomenon [4].

Chronic diseases are defined by the World Health Organization (WHO) [5] as disorders of "*long duration and generally slow progression*". The five major CD prioritized within the WHO program [5]: are cardiovascular disorders (CVD), cancer, chronic respiratory diseases, diabetes mellitus (DM) and mental disorders.

Over the last decade, increasing awareness on the nature of CD has triggered a new health paradigm that involves profound emerging changes both in healthcare practice and in the generation of biomedical knowledge. Three major distinctive traits, as compared with the traditional 20^th ^Century approaches, can be depicted:

**• Individual variability of disease expression **is being taken into account prompting a subject-centered approach both for disease characterization and patient management. It is acknowledged that identical causal factors may generate marked differences in disease expression at individual level. Those differences are only partly explained by the individual genetic background. It is increasingly accepted that the most important factors to explain differences among individuals are variations in pre- and post-translational biological regulation, as well as variability of the impact of life-style, environmental and behavioral factors on biological disease mechanisms.

**• Role of co-morbid conditions **- The occurrence of different chronic disorders in a given patient is common and co-morbidity has a well-recognized influence on high use of healthcare resources and poor prognosis. In this regard, the Charlson index [6] is clinically used in ICD-9 and ICD-10 administrative data as a marker of the impact of co-morbidity on prognosis; but other indexes do exist (e.g. Elixhauser Comorbidity Index) [7]. Moreover, patterns of highly prevalent CD (comorbidity clusters) [8], namely: cardiovascular disorders (CVD), chronic obstructive pulmonary disease (COPD) and type 2 diabetes mellitus (T2DM), hardly explained only by shared risk factors, are often observed [9]. Consequently, a better understanding and management of co-morbidity clusters constitutes a priority.

**• Integrated care with focus on preventive interventions**. Conventional reactive care strategies focusing on management of episodic crisis has shown to be inefficient in chronic care management [10-12]. Instead, prevention of exacerbations and cost-effective modulation of disease progress with a patient-oriented approach are two main drivers of the changes that are facing healthcare systems.

The Innovative Care for Chronic Conditions (ICCC) initiative [10-12], launched by the WHO in 2002, represented a major breakthrough in healthcare. Its merit was the formulation of the basic principles and strategies to improve management of patients with CD under the conceptual framework of the Chronic Care Model (CCM) promoting integrated care for chronic patients. However, the practicalities of its implementation and extensive adoption still remain a challenge.

Regarding the emerging changes in biomedical knowledge, on-going developments within the frame of network medicine [13,14] are setting the basis for a better understanding of CD mechanisms and co-morbidity clustering that will have a twofold impact. On the one hand, they will contribute to re-assessment of traditional disease taxonomies and to the formulation of a novel vision based on underlying patho-biological mechanisms of CD that will likely result in therapeutic innovations. On the other hand, these changes will foster Predictive Medicine [15,16] for chronic patients with relevant implications on management.

This scenario will result in enhanced subject-specific health-risk assessment providing the rationale for patient dynamic stratification that will support the design of cost-effective preventive strategies aiming at modulation of disease progress. The need for efficient and intensive use of Information and Communication Technologies (ICT) appears as a common requirement for deployment of integrated care, as well as to support further developments in biomedical knowledge of chronic disorders.

### Chronic obstructive pulmonary disease as a use case

Chronic obstructive pulmonary disease (COPD) is a prevalent chronic disorder included into the CD groups prioritized by the WHO that affects approximately 9% of adult population above 45 years [17]. The disease imposes a high burden on healthcare systems and it is currently the fourth cause of mortality worldwide [3,17].

COPD is caused by the inhalation of irritants, mainly tobacco smoking, in susceptible subjects: approximately 15 to 20% of all tobacco smokers. Pulmonary injury consist of inflammation, remodeling and destruction of lung parenchyma (emphysema) leading to progressive, only partially reversible, airflow limitation and, eventually, reduced arterial blood oxygenation.

Clinical manifestations of COPD begin in the adulthood, above 45 years, showing substantial individual variability of both clinical manifestations and pulmonary disease progression [18]. Patients may present acute episodes of severe exacerbations with a well demonstrated negative impact on prognosis [18]. COPD patients may also show systemic effects and co-morbid conditions both associated with poor prognosis [18,19].

Several reasons seem to support the selection of COPD as a use case to explore novel strategies to enhance knowledge on CD and to improve chronic patient management with an integrated care approach. First among these factors favoring the selection of COPD is its high prevalence and burden on health systems. A second reason is that the long-term COPD evolution may facilitate the assessment of the effects of preventive strategies on disease progression. Moreover, COPD heterogeneity, both in terms of disease manifestations and rate of disease progression [18] may pose specific challenges for subject-specific health risk assessment and healthcare stratification. As analyzed below, there is evidence indicating that COPD heterogeneity may follow a particular pattern with relevant implications on healthcare strategies for these patients. A final pragmatic reason for selecting COPD as a use case in the Synergy-COPD project is that recent deployment experiences of integrated care services in COPD patients [20,21] show high potential for generating effectiveness and cost-containment.

### The Synergy-COPD project

The general aim of Synergy-COPD was to explore the potential of using a computer-based systems medicine approach to improve knowledge on underlying mechanisms of COPD that should lead to better understanding of disease heterogeneity. The transfer of the biomedical knowledge generated during the project into healthcare aiming at enhancing chronic patient management was an intrinsic component of Synergy-COPD.

The classical approach to disease assumes that one or more causal factors trigger disease initiation often affecting one target organ or system. The individual variability of disease manifestations usually depends on: *i) *the intensity of exposure to the causal factor; and, *ii) *the interplay between a myriad of internal biological regulatory events and external factors (life style, environmental and behavioral elements) modulating disease manifestations. In this disease model, the rate of impairment of the target organ or system drives disease progression such that the systemic effects of the disease are explained the consequences of dysfunction of the target organ or system on the loss of whole-body homeostasis.

In COPD, the classical approach is illustrated by the *"spill over" *hypothesis [19,22] supporting a rather simplistic disease model that is far from explaining the complexities of COPD heterogeneity. According to the *"spill over" *hypothesis, pulmonary inflammation and remodeling caused by tobacco smoking generates inflammatory cytokines that leak into the blood stream being responsible for the low-grade systemic inflammation observed in some COPD patients [18,23,24] ultimately leading to systemic effects of the disease associated to poor patient prognosis [18]. This disease model only explains co-morbidity clustering through shared risk factors among concomitant disorders.

The current project was based on an alternative hypothesis relying on the assumption that COPD heterogeneity may not driven by the events occurring in the central organ, that is, the lung, as claimed by the classical "spill over" hypothesis [19,22]. Instead, COPD heterogeneity should be explained by the interplay of events occurring at three different levels: *i) Pulmonary disease *- determined by the effects of lung injury and local remodeling processes; *ii) ***Systemic effects of the disease** with different manifestations, being skeletal muscle dysfunction/wasting one of the most representative [25,26]; and, *iii) ***Co-morbidity clusters (patterns)** that refers to observed common associations of different chronic disorders.

The project used a systems approach to explore underlying mechanisms of COPD heterogeneity focusing on the systemic effects of the disease and the co-morbidity clustering. The research on pulmonary events was rather marginal in Synergy-COPD. The central hypothesis of the project was that systemic effects of COPD and co-morbidity clustering may share abnormal regulation of pivotal pathways leading to a progressive loss of integrative functions of the whole-body system as disease progresses [27]. In this scenario, it is hypothesized that nitroso-redox disequilibrium plays a relevant role in COPD progression [28,29].

As indicated above, the aims of Synergy-COPD were not only to generate biomedical knowledge, but also, to examine applicable strategies for a quick transfer of biomedical research novelties into the clinical practice. To this end, different modeling approaches were used as supporting tools aiming at covering three different aspects: *i) *to conceptualize and better understand underlying biological mechanisms of the phenomena targeted for study; *ii) *to identify (and possibly combined) biomarkers with potential predictive power; and, *iii) *to elaborate subject-specific predictive modeling for patient stratification in the clinical arena.

### Objectives and structure of the current manuscript

**Figure 1:**
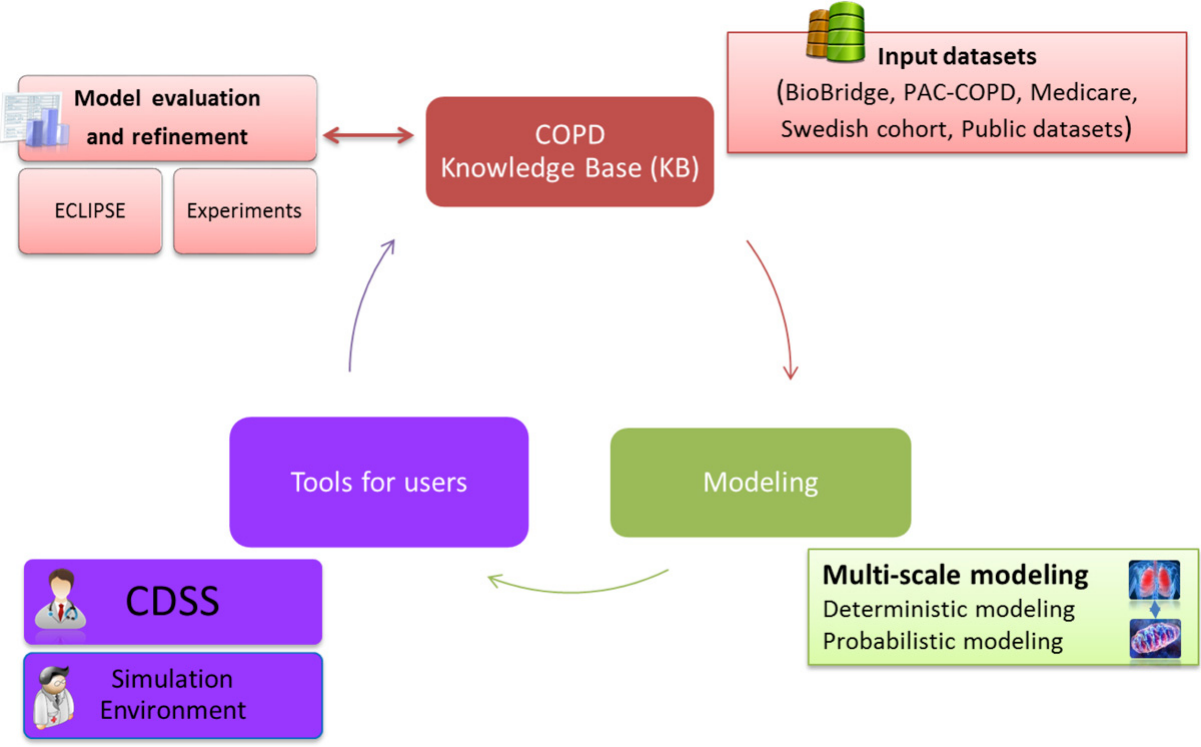
The Synergy-COPD translational research design.

This first chapter of the Supplement describes the aims, the overall design (Figure 1) and the different dimensions tackled in Synergy-COPD emphasizing its multidisciplinary nature. The entire Supplement has been conceived to reflect in detail the process of generation of the project and the complexities of its development. We hope that the different chapters will serve as a guide for other researchers aiming at generating novel biomedical knowledge using the potential of a systems approach, but also willing to transfer knowledge into innovative practices generating an iterative process between systems-oriented biomedical research and healthcare ultimately leading to enhanced cost-effective health outcomes. It is our purpose to drive the interested reader through the different papers associated to the Supplement aiming at providing insight into the different (but highly linked) aspects of the project and its outcomes. For these purposes, the Supplement is structured in four well-defined sections:

**• Section 1** - Presentation and overview of the project *(Chapters 1 and 2 *([30]* and this article)*

**• Section 2** - Addressing heterogeneity of chronic diseases *(Chapters 3 to 5 *[31-33]*)*

**• Section 3** - Tools and resources *(Chapters 6 to 8 *[34-36]*)*

**• Section 4** - Translation into the Clinical Field *(Chapters 9 to 11 *[37-39]*)*

The first two sections together with the last chapter (*Chapter 12 *- Challenges and opportunities [40]) provide general information on the outcomes of the project and identified gaps and perspectives for further developments beyond the project life-span. Section 3 covers the description of the tools through which the models have been made accessible, the resources required for their development, and the overall digital health framework where those tools and resources fit. Finally, Section 4 gives the details on the deployment of the research and tools made available to both the scientific and medical community and addresses three basic elements needed to ensure a successful deployment and adoption of 4P Medicine [41-43], that is Predictive, Preventive, Personalized and Participatory. Models for Systems Medicine require handling with specific data repositories covering a wide range of data types. For this, data exchange and data integration becomes crucial for a proper analysis, and the development of accurate models. In this fourth Section, Chapter 9 [36] depicts practical developments of Clinical Decision Support Systems (CDSS) embedded into clinical processes with the twofold purpose of bringing novel knowledge into clinical practice and supporting health professionals in the clinical decision making process. Chapter 10 includes the case study of a system to support distributed data management and data exchange for a Digital Health Framework [37] wherein a systems approach for interoperability among informal care, formal healthcare and biomedical research is provided. Finally, Chapter 11 describes a specific example of preparation of the workforce as a key element for building-up 4P Medicine [38]. The Chapter also indicates the current trends and strategies for workforce preparation at EU level.

## Project design and main components

Figure 1 depicts the overall project design covering the three main dimensions of Synergy-COPD, that is: *i) *Generation and validation of novel biomedical knowledge to better understand disease heterogeneity using a systems approach; *ii) *Development of novel technological supporting tools needed for the purposes of the project; and, *iii) *Design and validation of strategies to transfer biomedical knowledge into integrated care for chronic patients.

### Generation of novel biomedical knowledge on COPD heterogeneity

The basic descriptive features of the input datasets used to explore the different biomedical dimensions of the project, as well as those used for validation purposes, are displayed in Table 1. Both analyses and main outcomes of the biomedical aspects of the project are reported in detail in Chapter 3 on COPD heterogeneity [31]. Briefly, the project performed studies in three main areas:

***Skeletal muscle dysfunction ***- A network medicine approach was used to assess underlying mechanisms of skeletal muscle dysfunction and muscle wasting [44] by exploring the interactions among pathways modulating protein balance and tissue remodeling; cellular oxygenation & bioenergetics; insulin-resistance; with particular emphasis on the role of nitroso-redox disequilibrium. Moreover, the determinants of abnormal training-induced adaptations reported in COPD patients, as well as their potential impact on training protocols and recommendations of physical activity were addressed.

**Table 1:** Data sources used to explore the different biomedical dimensions of the project and for validation purposes.

Data-sets	Data-types	Reference	**Brief Description**.
**BioBridge**	*Metabolomics, Proteomics, Transcriptomics, Physiological and Clinical Measurements*	[27], [83], [84],	COPD patients with low and normal fat free mass index and healthy subjects studied before and after training.

**PAC-COPD**	*Clinical Measurements and Co-morbidity data*.	[75]	COPD patients studied after the first hospitalization because of an episode of exacerbation.

**Medicare**	*ICD9 codes from hospital registries*.	[85], [85]	Health Insurance Program

**Animal experiments**	*Muscle Transcriptomics and muscle & blood Cytokines*. Role of eiF6 in energy metabolism	Synergy-COPD-FP7-ICT-270086:2010-13.	Guinea Pig and mice models, C2C12 cell culture. Normoxic and hypoxic conditions

**Public datasets**	*SNOMED-CT, MeSH, angiogenesis-related expression data, etc*.	See **Table 1 **in COPDKB [35]	Public resources integrated into the Synergy-COPD Knowledge Base.

**Co-morbidity analyses **- An initial data driven approach aiming at assessing the relative risk for co-morbidity clustering in COPD patients was done. The research also identified genes and pathways associated with clusters of co-morbidities. The outcomes were compared with the analysis of targeted co-morbidities often seen in COPD patients: cardiovascular disorders (CVD) and Type 2 Diabetes Mellitus-Metabolic syndrome (T2DM-MS) that were identified through different reports [45-48]. The relevant pathways identified in the analysis of the systemic effects of COPD were compared with those seen in the co-morbidity clustering to explore commonalities.

**Pulmonary events **- The project addressed the analysis of the dissociation between low central airways resistance and high emphysema score seen in approximately one third of the patients from the PAC_COPD study [23]. To this end, we used the lung modeling techniques described in detail in Chapter 5 generated in collaboration with the AirPROM project [49].

### Development of novel technological supporting tools needed for the purposes of the project

Several ICT-supporting tools were developed to cover the requirements throughout the project life-span. A detailed description of these technological components is carried out through Chapters 4 to 10. Each of the items described in this subheading, and subheading 2.3, plays a relevant role and their articulation was pivotal for the project development and the generation of the expected outcomes.

**Synergy-COPD Knowledge-Base **A first version of the COPD Knowledge Base (COPD-KB) was developed as part of the Biobridge project to answer specific queries on COPD mechanisms [50]. The COPD-KB consists of an object-oriented semantic data integration approach to allow researchers - by a user-friendly *query wizard *- to create *complex semantic networks of relationships*. Briefly the COPD-KB stores elements (e.g. genes, disease, proteins among many others) and it connects a pair of elements if they are found associated by direct evidence (*e.g. *protein-protein interactions) or indirect evidence (e.g. two genes that have been associated to the same pathway) that can be generated by semantic mapping. The initial COPD-KB was used as a backbone to create a new version which: (1) updates the existing data-bases and ontologies; (2) it incorporates novel ones (e.g. disease ontologies such as ICD9); and, (3) it includes the different network models obtained during the lifetime of Synergy-COPD from data of COPD projects. All-in-all the COPD-KB, described in detail in Chapter 6 [34] represents the status of the art in COPD knowledge management and has been widely used as a required resource in the development of novels tools for System Medicine [32], and to provide support to the Simulation Environment (by storing all information on models in a easily accessible manner) [35].

**Computer modeling approaches**. We define a model as *the description of a system by describing its elements and their interactions*. These descriptions can be *qualitative *and/or *quantitative*. A classical example of a qualitative model is a protein-to-protein interaction network which are, frequently used in network medicine; the links of those networks are gathered through experimental observations or predicted by computational methods [51]. A typical example for a quantitative model is the description of a chemical reaction system via ordinary differential equations. The two types of models offer different —and often complementary— insights into a system, each requiring its own set of mathematical tools [13,52-55]; see Chapter 4 [32] for more detailed examples.

The Synergy-COPD project integrated existing physiological quantitative modeling of O_2 _transport and utilization [56-58] with biochemical modeling of mitochondrial reactive oxygen species (ROS) generation [59] providing a quantitative estimation of the relationships between cellular oxygenation and ROS levels, as explained in detail in Chapters 3 [31] and 4 [32]. The project also aimed at exploring the functional impact of spatial pulmonary heterogeneity, as reported in Chapter 5 [33].

Synergy-COPD also produced different modalities of network modeling aiming at exploring targeted biomedical questions. Moreover, the project generated interoperability tools between deterministic modeling and different modalities of network analysis, as extensively described in Chapter 4 [32]. Synergy-COPD has evidenced the potential of the current modeling tools, but also their limitations and the directions for further developments [39].

Specific project outcomes were generated using modeling to better understand biological mechanisms of the disease. However, due to different factors, the validation of combined biomarkers and the generation of subject-specific predictive modeling could not be addressed during the project lifetime, as initially planned. Despite of these limitations, one of the main outcomes of the project was the design and validation of pragmatic strategies for transferring novel biomedical knowledge into the clinical arena. To this end, the results of the biomedical research carried out in the project, together with existing knowledge, were used to generate rules feeding clinical decision support systems (CDSS) for early disease detection, for patient health risk assessment and stratification, and for community-based enhanced patient management, as reported in Chapter 9 [36]. The CDSS produced in the project were embedded into clinical processes supported by an Integrated Care Shared Knowledge Platform [20,60] and specific validation strategies were defined and executed.

**COPD Simulation Environment**. The use of modeling in basic research has been growing in the last decades. Tools, such as COPASI [61] or Chaste [62], and standards, such as SBML [63] or CellML [64], have allowed model sharing and simulation reproducibility fostered by model repositories such as Biomodels [65] or CellML model repositories [66]. Despite the increasing number of tools, the resources are not yet being used in clinical research due majorly to complexity of their management. Therefore, the Synergy-COPD project prioritized to develop environments where clinicians may make use of models and may start running simulations on their own. To this end a Simulation Environment (SE) was developed to host a set of preselected mechanistic and probabilistic (Bayesian networks) models ([35,32]) that are associated to COPD-related physiological processes; models were coded in different programming languages (*e.g. *C++ and Fortran). The SE is composed of a web interface (graphical visualization environment) and a simulation workflow management, allowing the user to run and store simulations. Furthermore the SE allows running simulation of several models consecutively by feeding the output of model A as input of model B. The SE connects to the Synergy-COPD KB [34] in order to gather information of the quantitative models such as parameter values, ranges and names.

### Design and validation of strategies to transfer biomedical knowledge into integrated care for chronic patients

Computer modeling approaches as described above refer to their use to explore biological disease mechanisms aiming at understanding CD, as well as to identify combined biomarkers with potential for characterizing subject variability and/or with predictive value. It is of note, however, that subject-specific computer-based modeling including different categories of covariates (biological, physiological, clinical, behavioral, etc.) can be also useful as predictive tools for clinical purposes. De facto, rules derived from such modeling approaches should be expected to feed CDSS embedded into clinical processes.

The reality, however, is that current modeling tools for health risk assessment [31] rely on population-based analyses of past use of healthcare resources. These predictive tools are useful to support interventions and/or to define health policies at group level, but they show limitations for clinical applicability at patient level.

**Figure 2:**
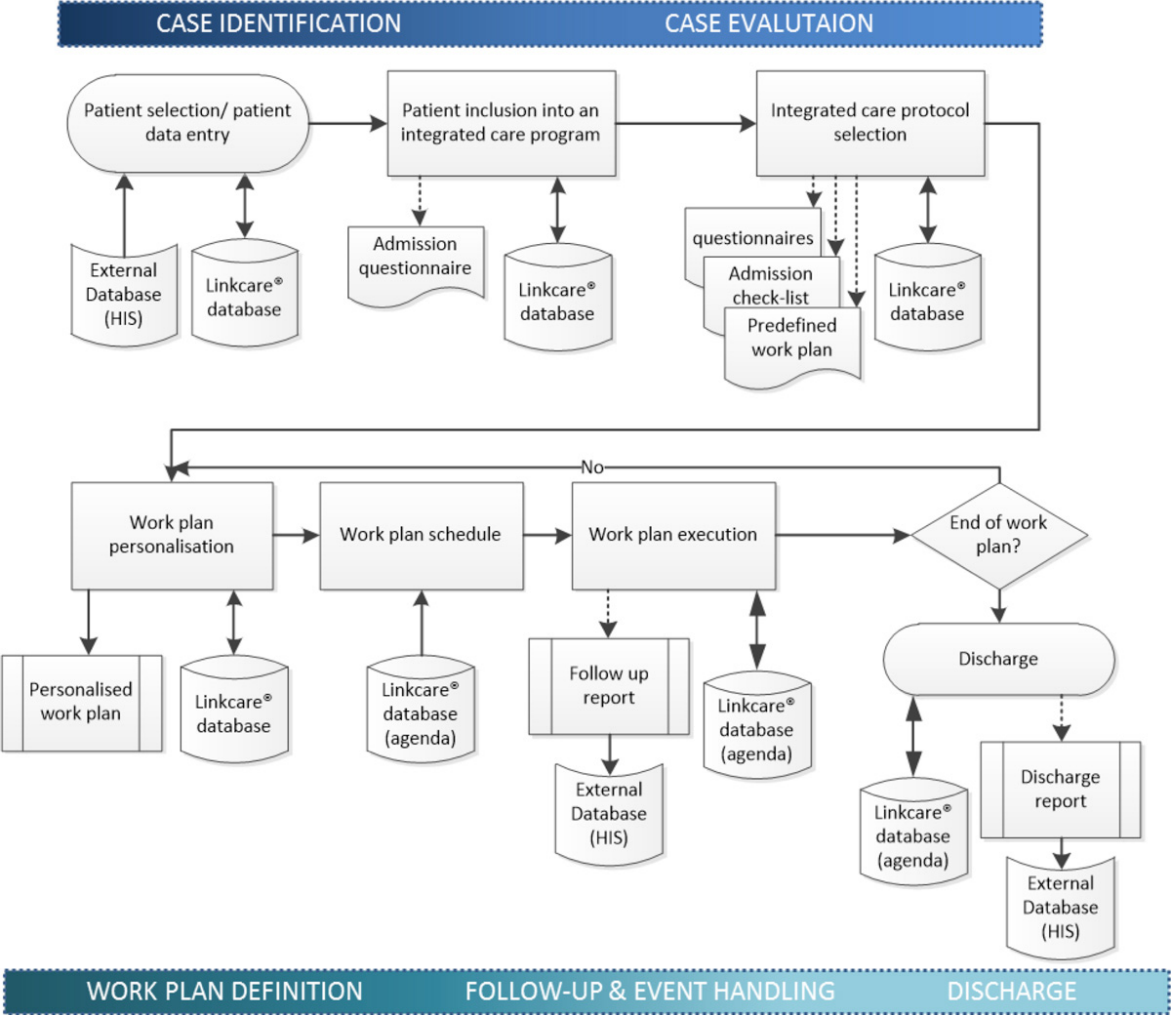
**Workflow for an Integrated Care Service (ICS) management and execution**. The patient is allocated into an ICS defined as a set of well standardized tasks to be carried out on the basis of his/her health condition and social circumstances to achieve target objectives aligned with the comprehensive treatment plan. Two differential ICS characteristics, as compared to usual care, are the patient-centered approach and the longitudinal nature of the interventions which length depends on the type of ICS. The corresponding ICS template is used as a library of resources for the correct customization and execution of the ICS workflow. Integration with corporate Electronic Health Record (EHR) allows instant access to required patient information for case identification and evaluation, avoid data duplicity and preserve the current corporate clinical data chain of custody. Follow-up and discharge reports can also be sent to the corporate EHR to keep trace of the ICS execution as part of the patient clinical episodes.

One of the Synergy-COPD goals was to facilitate elaboration of refined subject-specific health risk assessment with a holistic patient-oriented approach. Such modeling approach constitutes the basis for 4P medicine facilitating the design of early interventions with a preventive orientation. Moreover, the prediction of health risk should contribute to patient-based stratification in the healthcare arena, as indicated in Figure 2 wherein the logical sequence for chronic care management is displayed [20,21].

**Figure 3:**
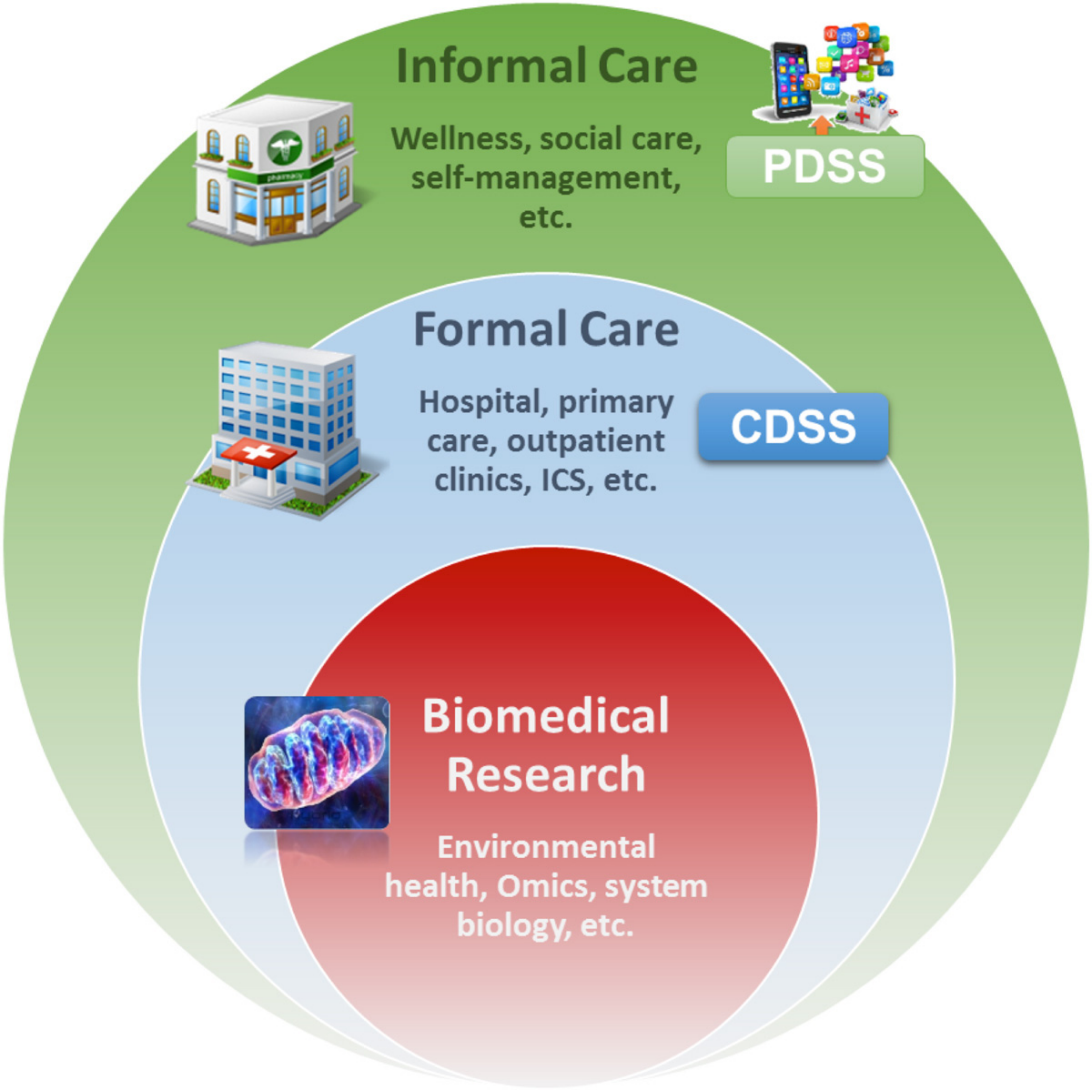
**Patient information collected through the Personal Health Folder (PHF, Informal Care) and through the Electronic Health Record (EHR, formal healthcare) will be used to feed the biomedical research platform wherein subject-specific predictive modeling will be generated using the knowledge-base and tools for data analysis**. Predictive modeling will generate rules to feed Clinical Decision Support Systems (CDSS) embedded into clinical processes to support health professionals. Moreover, it will also help to generate Patient Decision Support Systems (PDSS) embedded into the PHF aiming at patient empowerment for self-management of his/her condition. Interoperability of the three steps indicated in the figure constitutes the Digital Health Framework (DHF) extensively described in Chapter 10 [37].

The project hypothesizes that a systems analysis of COPD heterogeneity may facilitate the identification of combined biomarkers with predictive power of disease progress. The elaboration of subject-specific predictive modeling as initially addressed in the project only relied on the analysis of biological phenomena, but further enrichment with other types of input data, namely: patient adherence profile, life-style, clinical and social factors involving frailty risk, etc...) must be considered, as shown in Figure 3. Efforts on personalized modeling, such as the integrated use of bayesian and mechanistic models, are described in Chapter 4 [32].

The project adopted a pragmatic approach to the challenge by identifying the following major steps in the process of knowledge transfer into the clinical scenario: *i) *validation of the biomedical findings using independent datasets from human and animal studies, as described in detail in Chapter 3 [31]; *ii) *formulation of CDSS based on knowledge generated within the project and enriched with existing knowledge, see Chapter 9 [36] for further details, *iii) *qualitative evaluation of the CDSS using a focus group strategy that includes different profiles of healthcare professionals, and, *iv) *Integration of the CDSS into an Integrated Care Shared Knowledge Platform [20,21,60] supporting the clinical processes. The clinical impact of the CDSS shall be assessed beyond the project lifetime.

In Synergy-COPD, we aligned the development of a CDSS with the following goals: (1) early detection and diagnosis of COPD coordinating informal care (pharmacy offices) and primary care, (2) spirometry quality control support, (3) patient health risk assessment and stratification and (4) therapy recommendation, as described in detail in Chapter 9 [36].

## Systems medicine and expected contributions of Synergy-COPD

It is extensively accepted that the complexity of life cannot be untangled by investigating it from a reductionist point of view. Indeed, biological systems tend to show *emergent *behaviors, i.e. the whole system is more complex than the sum of its individual *parts. Systems biology *(SB) explores these emergent phenomena by studying not only the elements *of a system*, but more importantly their *interactions*. The origins of SB in 2001 [67] and its posterior developments in *different *areas, namely: biochemistry (*e.g. *quantitative modeling of enzyme kinetics) [68], mathematics (*e.g. *control theory) [69], and neuroscience (*e.g. *Huxley-model) [70] have generated the basis of the discipline during the last decade. From this diverse background a very rich and heterogeneous field has evolved for which a single, universal definition is difficult. Perhaps it is this very ability to defy traditional boundaries that enabled SB to successfully apply transversal methods from physics to biology [53,54]. The use of a systems approach in medical research constitutes *Systems Medicine *(SM) [71] and its application into the healthcare *conforms Predic*tive Medicine [41,72], one of the components of 4P Medicine.

As indicated above, COPD is certainly a target for a systems approach. The classical definition of COPD - poorly reversible airflow obstruction [17] - is being questioned from a *systems *perspective [73]. It is not operational to properly address COPD heterogeneity [24,45,74] and the boundaries with other obstructive airway diseases [75] are poorly defined prompting the need for new disease taxonomies based on underlying mechanisms with impact on clinical management [31]. Recent state-of-the-art SB/SM research on COPD has provided exciting results indicating a high potential for the approach. For example, a systems re-analysis of lung gene expression data comparing COPD and healthy individuals [76] identified several affected pathways pointing toward potential novel drug targets [77,78]. A second example is given by a new approach combining gene expression and clinical data to identify subgroups of COPD patients [79]. In a third example relations between COPD phenotypes are described by networks; the approach allowed the identification of novel phenotype-phenotype associations [80]. Last, but not least, the Biobridge project provided pivotal results to substantiate the central hypothesis of the current project.

In Synergy-COPD, we aim to develop models that may have an impact on both biomedical research and clinical management of chronic patients. The later is expected to be achieved by generating model-derived rules feeding the CDSS embedded into the clinical processes. To this end we considered to approach systems medicine in COPD as a two-stage process. *Stage 1 *aims at identifying of mechanisms and relevant markers of systemic effects of COPD and COPD co-morbidity clustering combining the use of deterministic modeling and network medicine approaches. Within this first stage, the CDSS were developed combining existing knowledge with knowledge acquired during the project, but ICT tools supporting the linkage between subject-specific predictive modeling and CDSS generation could not be developed. In *Stage 2*, to be developed beyond the project life span, refined subject-specific predictive modeling combining different categories of covariates, as described above, should be developed and properly validated. Interestingly, this approach can be considered a case study of *networks for advanced Decision support *[81].

## Summary

The current manuscript acknowledges the dramatic societal impact and the challenges posed by the epidemics of CD. The selection of COPD as a use case representing the complexities of different chronic disorders is justified. Moreover, the basics of the Synergy-COPD project design are presented. We highlight the potential of a systems approach to address the COPD challenges and we define strategies for transferring biomedical knowledge into the healthcare scenario proposing an ecosystem able to support productive iterations between biomedical research and healthcare. One of the purposes of the chapter is to introduce the reader into the description of goals, resources and methodologies generated by Synergy-COPD, and to provide a guide for the reading of all them as reported in the different manuscripts associated to the Supplement.

## Competing interests

The authors declare they have no competing interests.

## Declaration

Publication of this article has been funded by the Synergy-COPD European project (FP7-ICT-270086). The opinions expressed in this manuscript are those of the authors and are not necessarily those of Synergy-COPD project's partners or European. The

This article has been published as part of *Journal of Translational Medicine *Volume 12 Supplement 2, 2014: Systems medicine in chronic diseases: COPD as a use case. The full contents of the supplement are available online at http://www.translational-medicine.com/supplements/12/S2.
